# Atorvastatin pretreatment diminishes the levels of myocardial ischemia markers early after CABG operation: an observational study

**DOI:** 10.1186/1749-8090-5-60

**Published:** 2010-08-13

**Authors:** Erdal Ege, Yüksel Dereli, Sevil Kurban, Ali Sarigül

**Affiliations:** 1 Selçuk University, Meram Medical School, Department of Cardiovascular Surgery, Konya, Turkey; 2 Konya Numune Hospital, Department of Cardiovascular Surgery, Konya, Turkey; 3 Selçuk University, Meram Medical School, Department of Biochemistry, Konya, Turkey

## Abstract

**Background:**

Statin pretreatment has been associated with a decrease in myocardial ischemia markers after various procedures and cardiovascular events. This study examined the potential beneficial effects of preoperative atorvastatin treatment among patients undergoing on-pump CABG operation.

**Methods:**

Twenty patients that had received atorvastatin treatment for at least 15 days prior to the operation and 20 patients who had not received any antihyperlipidemic agent prior to surgery were included in this study. CK-MB and troponin I levels were measured at baseline and 24 hours after the operation. Perioperative variables were also recorded.

**Results:**

Twenty-four hours after the operation, troponin I and CK-MB levels were significantly lower in the atorvastatin group: for CK-MB levels, 12.9 ± 4.3 versus 18.7 ± 7.4 ng/ml, p = 0.004; for troponin I levels, 1.7 ± 0.3 versus 2.7 ± 0.7 ng/ml, p < 0.001. In addition, atorvastatin use was associated with a decrease in the duration of ICU stay.

**Conclusions:**

Preoperative atorvastatin treatment results in significant reductions in the levels of myocardial injury markers early after on-pump CABG operation, suggesting a reduction in perioperative ischemia in this group of patients. Further studies are needed to elucidate the mechanisms of these potential benefits of statin pretreatment.

## Background

Ambulatory use of 3-hydroxy-3-methylglutaryl-CoA (HMG-CoA) reductase inhibitors, or statins, is known to reduce the risk of cardiovascular events including death, myocardial infarction, stroke, and renal function, in addition to their lowering effect on low-density lipoprotein (LDL) and total cholesterol levels [1]. However, beneficial effects of statin treatment are not limited to the patients with hypercholesterolemia. Patients with normal or low levels of LDL also benefit from long term statin treatment with lower incidence of cardiovascular events and reduced need for coronary angioplasty or coronary surgery [2].

Cardiac isoforms of troponin are specific markers for myocardial injury. They are highly sensitive indicators for perioperative myocardial ischemia [3]. Elevated levels of troponin following revascularization procedures like percutaneous coronary interventions and coronary artery bypass grafting (CABG) have been associated with increased risk of cardiac complications [4]. Even after a successful percutaneous coronary intervention, 5 to 30% of patients experience elevations of cardiac biomarkers [5]. Among stabile angina patients that underwent elective coronary intervention, administration of atorvastatin for 7 days before the procedure has been shown to reduce procedure-related myocardial injury substantially [6].

This study examined the potential beneficial effects of preoperative atorvastatin treatment given for at least 15 days before on-pump CABG on myocardial injury indicators, CK-MB and troponin I.

## Methods

### Patients

Forty patients undergoing elective CABG were included in this study. Twenty consecutive patients that had received minimum 20 mg/kg/day atorvastatin (Ator, Sanovel, Istanbul, Turkey) for at least 15 days before surgery constituted the study group and 20 consecutive patients that had not received any antihyperlipidemic agent prior to surgery were included in the control group. Exclusion criteria were as follows: valvular repair or any additional cardiac procedure, COPD, left ventricular ejection fraction <30%, emergency operations, and severe hepatic or renal failure (creatinine > 2 mg/dl). The study protocol was approved by the Ethics Committee of Selcuk University Meram Medical Faculty.

### Surgical method

All patients underwent primary CABG operation using standard cardiopulmonary bypass. Fentanyl, midazolam and pancuronium bromide were used for the induction of anesthesia. Median sternotomy was used for all operations and vascular conduits were prepared before the commencement of cardiopulmonary bypass. Then 300 IU/kg heparin was administered and cardiopulmonary bypass with a roller pump was initiated under moderate hypothermia using standard aortic and two-stage venous cannula. Cold blood cardioplegia was used in all patients. Preoperative and postoperative parameters including durations of aortic cross clamp, respiratory support, ICU stay, and hospitalization were recorded as well as pulmonary parameters (arterial blood gas analysis) and the need for inotropic agents. In addition, serum LDL cholesterol level, erythrocyte sedimentation rate and leukocyte count were recorded preoperatively.

### Measurements of troponin I and CK-MB levels

Blood samples for biochemical analyses were obtained at the time of anesthesia induction and 24 hours after the operation from right radial artery. They were kept at room temperature for 30 minutes before they were centrifuged at 3000 rpm for 5 minutes to separate sera (Eppendorf centrifugation device 5840; Eppendorf, Hamburg, Germany). All blood samples were stored at -80°C until analysis.

Serum troponin I levels were measured by a commercially available chemiluminescent immunoassay on an autoanalyser (Immulite Diagnostic Products Co., Los Angeles, CA, USA). For the quantitative measurements of serum CK-MB levels, a commercially available chemiluminescent enzyme labeled immunometric assay was used on an autoanalyser (Immulite Diagnostic Products Co., Los Angeles, CA, USA).

#### Statistical analysis

Statistical analysis was performed using SPSS version 15.0 software (SPSS Inc., Chicago, IL, USA) for Windows. Continuous variables were expressed as mean ± SD or median and interquartile range. Differences between groups were tested using Student t test or Mann-Whitney U-test. Categorical data were compared using Chi-square test or Fisher's exact test. A p value < 0.05 was considered as an indication of statistical significance.

## Results

Demographical, clinical and operative data of the two groups are presented in Table 1. The two groups did not differ with regard to age, gender, weight, preoperative laboratory findings, cardiovascular risk factors, and perioperative variables. No statistically significant difference was found in LDL levels between the two groups. Transient atrial fibrillation developed in one patient in each of the groups (p = 1.00) and no other arrhythmia was observed in any of the subjects.

**Table 1:** Demographical, clinical and operative data of the patients (n = 40)

Characteristics	Atorvastatin pretreatment n = 20	No atorvastatin pretreatment n = 20	P for difference
***Demographical and baseline clinical data***			

Age, y	58.8 (8)*	61 (11) *	0.44

Weight, kg (mean ± SD)	83.1 ± 2.4	83.6 ± 2.3	0.88

Male to female ratio	16/4	15/5	1.0

Diabetes, n (%)	7 (35%)	9 (45%)	0.51

Hypertension, n (%)	6 (30%)	6 (30%)	1.0

Ejection fraction, %	40 (8.5)*	40 (13.5)*	0.26

LDL, mg/dl (mean ± SD)	100.7 ± 9.20	97.5 ± 6.0	0.20

CK-MB, ng/ml	2 (1.5)*	1.85 (1.6)*	0.79

Troponin I, ng/ml	0.2 (0.2)*	0.2 (2.6)*	0.30

Erythrocyte sedimentation rate, mm/h (mean ± SD)	18.9 ± 7.3	18.3 ± 6.8	0.77

Preoperative creatinine level, mg/dl	1.0 (0.3)*	1.0 (0.28)*	0.47

***Intraoperative and postoperative parameters***			

Duration of aortic cross clamp, min (mean ± SD)	63.5 ± 20.8	65.3 ± 20.9	0.79

Duration of CPB, min (mean ± SD)	103.2 ± 30.7	98.3 ± 26.3	0.58

ICU stay time, d	2.0 (1.0)*	3.5 (2.5)*	0.046

Duration of intubation, h	8.0 (5.5)*	7.0 (10.25)*	0.968

PO2, mmHg	93.8 (22.93)*	86.0 (20.99)*	0.26

CO2, mmHg	36.45 (3.64)*	37.8 (3.67)*	0.25

SaO2, %	97.0 (3.75)*	95.4 (3.25)*	0.686

Inotropic support, n (%)	10 (50%)	13 (65%)	0.33

Duration of hospitalization, d	7.0 (1.0)*	8.0 (1.75)*	0.25

Number of bypasses, n	3.0 (1.75)*	3.0 (1.0)*	0.14

Postoperative creatinine level, mg/dl	1.1 (0.48)*	0.95(0.6)*	0.37

Need for blood and blood products, U	3.5 (1.0)*	4.0 (1.0)*	0.97

Postoperative EF, %	40 (10.25)*	42 (10.25)*	0.94

Total postoperative bleeding, ml	762.5 (298.7)*	775 (267.5)*	0.82

* median (interquartile range).

**Figure 1:**
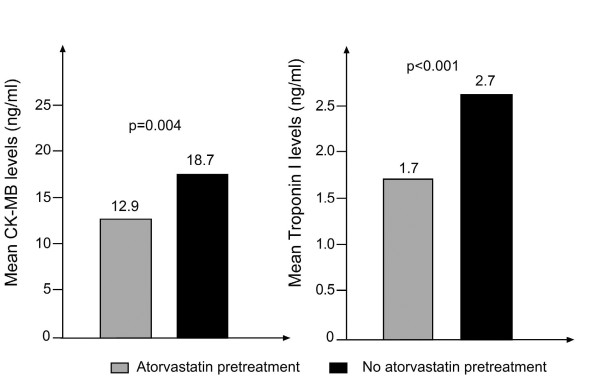
**CK-MB (A) and troponin I (B) and levels of the patients 24 hours after CABG operation**.

Although troponin I and CK-MB levels were similar at baseline (Table 1), 24 hours after the operation both levels were significantly lower in the group that had received atorvastatin compared to controls: for CK-MB levels, 12.9 ± 4.3 versus 18.7 ± 7.4 ng/ml, p = 0.004; for troponin I levels, 1.7 ± 0.3 versus 2.7 ± 0.7 ng/ml, p < 0.001 (Figure 1). Groups did not differ with regard to postoperative variables, except for a shorter duration of ICU stay among patients that had received atorvastatin pretreatment (p = 0.046) (Table 1). Early mortality was not observed in either of the groups.

## Discussion

The main finding of this study is the decreased early postoperative levels of myocardial injury indicators in association with the use of atorvastatin for a certain period prior to the CABG operation. In addition, atorvastatin treatment was associated with shorter duration of ICU stay. Preoperative statin use seems to have a role in decreasing CABG associated morbidity through attenuation of cardiopulmonary bypass-related acute inflammatory reaction and improvement of endothelial function owing to its antioxidant activities.

Beneficial effects of statin pretreatment have already been demonstrated in a number of studies on patients undergoing cardiac interventions. In a randomized study, pretreatment with atorvastatin before angioplasty has been shown to decrease the incidence of myocardial injury when compared to placebo. Atorvastatin pretreatment was associated with a significant reduction in the release of all myocardial injury indicators like myoglobin, troponin I, and CK-MB following the percutaneous procedure [5]. In another study with a design similar to this study, except for the use of a different statin and placebo, Mannacio et al. administered one-week 20 mg/day rosuvastatin treatment or placebo before CABG operation and found significantly lower levels of troponin I, myoglobin and creatinine kinase in association with rosuvastatin treatment compared to placebo, indicating less prevalent perioperative myocardial injury [7]. Similar to the findings of these previous studies, this study found lower levels of troponin I and CK-MB in association with preoperative atorvastatin use among patients undergoing CABG, providing further evidence for the benefits of statin administration for a period prior to coronary interventions.

Besides, several studies confirmed the clinical benefit of statins in terms of reduced mortality and morbidity. Preoperative statin treatment was shown to decrease 30-day mortality and acute MI risks significantly after CABG [8]. Likewise, in the retrospective study by Magovern et al. on 2377 patients, decreased operative mortality rates was evident among high-risk patients in association with preoperative statin treatment [9]. Long-term benefits of statin treatment have also been shown after CABG operation. Aggressive lipid lowering therapy has been shown to slow down progression of obstructive changes in saphenous vein grafts and reduce the need for a new revascularization procedure [10]. Significantly lower 30-day MI and mortality rates were observed among acute coronary syndrome patients if they were on statins at the time of the event [11]. Although not the subject of this study, current evidence suggest that ischemia preventing effect of atorvastatin during perioperative period may well translate into or contribute to longer term benefits with continued use.

Based on this growing evidence, initiation of statin treatment at the time of revascularization planning has become a widely accepted practice. Although the optimal duration of pretreatment to obtain clinical benefit is not yet clear, experimental data suggest that 14 days of pretreatment would have substantial favorable effect on inflammation and endothelial function [12]. Therefore, patients that had received at least 14 days of atorvastatin treatment were included in the study group of the present study.

Cardiac isoforms of troponin are specific myocardial injury markers indicating the level of perioperative myocardial ischemia. Moderate elevations of troponin I and T after CABG operation suggests minimal and reversible injury [3]. Troponin I is more sensitive than CK-MB and troponin T for the assessment of myocardial injury [13]. Although clinical implications of troponin I release after coronary interventions have not been widely studied, observational studies have found a correlation between troponin I levels and untoward events during hospitalization. In contrast, normal troponin I levels after coronary procedures almost eliminate the risk for in-hospital complications [14]. Thus, the lower troponin I levels among the atorvastatin group compared to controls found in this study may translate into lower postoperative complication rates, both in terms of mortality and morbidity, which warrants investigation in long term randomized controlled trials.

Asymptomatic myocardial injury as assessed by elevations of CK-MB levels is quite frequent after coronary interventions with a prevalence ranging between 10 to 40% of the cases [15]. Only a small increase in myocardial necrosis indicators without any impairment of cardiac function or ECG change may be seen in most of the patients [16]. In this study, the levels of myocardial injury indicators troponin I and CK-MB were significantly lower at 24 hours after CABG procedure in patients that received preoperative atorvastatin treatment.

In the study by Kourliouros et al., statin treatment was associated with a lower incidence of atrial fibrillation and a shorter duration of hospitalization after cardiac surgery [17]. However, they did not find any change in the duration of ICU stay. In contrast, this study found a shorter duration of ICU stay associated with statin treatment and no difference in terms of postoperative atrial fibrillation and duration of hospitalization. Significant reduction in myocardial damage as demonstrated by low levels of indicators might indirectly contribute to the reduced need for ICU support. However, it is of note to emphasize that many factors may prolong ICU stay, and this study found only a marginal difference between the two groups in terms of duration of hospital stay (p = 0.046). Future studies with larger sample sizes allowing multivariate analysis to adjust for multiple confounding factors would provide robust evidence on potential effect of atorvastatin treatment on the duration of ICU stay or hospitalization. Increasing the number of patients would also probably result in sufficient number of incidences related to postoperative ischemia that would translate into prolonged ICU and/or hospital stay. Thus, until then, such a possible indirect effect of atorvastatin treatment should be interpreted cautiously.

Experimental and clinical studies suggest that beneficial effects of statins may be beyond their cholesterol lowering effect [18,19]. These pleiotropic effects independent of cholesterol lowering include the improvement of endothelial function, NO related antioxidant activity, and inhibition of inflammatory response, vasoconstriction, thrombosis, and thrombocyte aggregation [20]. Several studies demonstrated a decrease in systemic inflammatory response with statin treatment during on-pump CABG operations. Chello et al. demonstrated a decrease in P-selectin release from the endothelium and CD11b release from neutrophils after CABG with statin treatment, which in turn inhibits the adhesion of activated neutrophils to the vascular endothelium [21]. In addition, neutrophil apoptosis was increased and the levels of circulating adhesion molecules ICAM-1 and ELAM-1 were decreased. They also showed that protective effect of statins on vascular endothelium was evident even at doses ineffective for the reduction of cholesterol levels [21]. In a previous study, we found a decrease in cardiopulmonary bypass-related systemic inflammatory response and endothelial function improvement in association with preoperative atorvastatin treatment in patients undergoing elective CABG operation [22]. Using experimental ischemia and reperfusion model, preoperative statin treatment have been shown to augment cardioprotective effects, significantly reduce myocardial infarct area and preserve cardiac contractile function and coronary perfusion [23]. Recent studies showed that statins affect important factors taking part in the pathogenesis of acute coronary syndrome including endothelial NO, endothelin, metalloproteinases, plasminogen activating factor, tissue plasminogen activator, and free radical production. The molecular basis of these statin effects beyond cholesterol lowering is the inhibition of isoprenoid intermediate pathways of cholesterol metabolism [24]. Above mentioned anti-inflammatory and antioxidative mechanisms, and improved endothelial function all seem to be responsible for and contributing to the reduced ischemia associated with perioperative atorvastatin use, among patients undergoing CABG or other coronary interventions.

This study has several limitations. First, this study evaluated troponin I and CK-MB levels before and at 24 hours after the operation. If serial blood samples had been obtained instead of a single measurement after the operation, the course of myocardial ischemia under atorvastatin treatment could be evaluated with reference to the control group. Second, our sample size is relatively small. Greater number of enrolled patients would be associated with a reduction of a potential statistical type II error, particularly for parameters other than markers and ICU stay time, and multivariate analysis allowing adjustment for multiple factors would be possible. Finally, a randomized controlled design would provide robust evidence.

## Conclusions

In conclusion, findings of this study suggest that preoperative atorvastatin treatment results in a significant reduction in the levels of myocardial injury indicators among patients undergoing on-pump CABG operation, thereby providing a benefit in terms of reducing perioperative ischemia in this group of patients. This seems to be due to a reduction in acute inflammatory reaction and cardioprotective effects of statins through NO related antioxidant activity and improvement of endothelial function. Larger randomized controlled studies with robust design allowing adjustment for confounding variables would provide further insight into the benefits provided by statin pretreatment and their mechanism.

## List of Abbreviations

CABG: coronary artery bypass grafting; CK-MB: creatinine kinase-MB; ICU: intensive care unit; HMG-CoA: 3-hydroxy-3-methylglutaryl-CoA; LDL: low-density lipoprotein; COPD: chronic obstructive pulmonary disease; SPSS: Statistical Package for Social Sciences; MI: myocardial infarction; ECG: electrocardiogram; NO:nitric oxide; ICAM-1: intercellular adhesion molecule 1; ELAM-1: endothelium leukocyte adhesion molecule 1

## Competing interests

The authors declare that they have no competing interests.

## Authors' contributions

EE; has made substantial contributions to conception and design,YD: acquisition of data, SK: analysis and interpretation of data, AS: has been involved in drafting the manuscript or revising it critically for important intellectual content; All authors read and approved the final manuscript.
